# Association of age at antiretroviral therapy initiation with CD4^+^ : CD8^+^ ratio recovery among virally suppressed people with HIV

**DOI:** 10.1097/QAD.0000000000003801

**Published:** 2024-02-26

**Authors:** Clare J. Holden, Fiona C. Lampe, Fiona M. Burns, Clinton Chaloner, Margaret Johnson, Sabine Kinloch-De Loes, Colette J. Smith

**Affiliations:** a Institute for Global Health, UCL, London; b The Royal Free NHS Foundation Trust; c Institute of Immunity and Transplantation, Royal Free Hospital, London, UK.

**Keywords:** aging, antiretroviral therapy, CD4^+^ : CD8^+^ ratio, HIV

## Abstract

**Objective:**

To investigate the association of age at antiretroviral therapy (ART) initiation with CD4^+^ : CD8^+^ T-cell ratio in virally suppressed people with HIV on long-term ART, and to characterize potential CD4^+^ : CD8^+^ ratio recovery in this population by age.

**Design:**

A longitudinal study of people attending an HIV clinic at the Royal Free Hospital NHS Trust, London, who initiated ART between 2001 and 2015, and achieved and maintained HIV-1 viral suppression (viral load <1,000 copies/ml). The association of age group at ART initiation with CD4^+^ : CD8^+^ ratio at 5 and 10 years was assessed.

**Methods:**

Multivariable linear regression was used to investigate the relationship between age at ART initiation and log CD4^+^ : CD8^+^ ratio, adjusting for demographic factors (gender/HIV transmission route, ethnicity), baseline CD4^+^ count and calendar year.

**Results:**

The sample included 1859 people aged 20–78 (75% men, 56% white ethnicity). Overall, median CD4^+^ : CD8^+^ T-cell ratio increased from 0.24 at baseline to 0.77 at year 5 and 0.88 at year 10. Ratios increased among all age groups in unadjusted and adjusted models but increased less among older ages (baseline ages 60–69 and 70–79). Median ratios at year 5 were 0.85, 0.80, 0.72, 0.76, 0.6, and 0.44, respectively, among people aged 20–29, 30–39, 40–49, 50–59, 60–69 and 70–79 years at baseline.

**Conclusion:**

In a virally suppressed London population, age had a substantial impact on CD4^+^ : CD8^+^ ratio recovery, especially for those starting ART after age 60 years. Results may indicate the level of CD4^+^ : CD8^+^ ratio recovery possible in an HIV-positive, virally suppressed, aging population.

## Introduction

The population of people with HIV (PWH) is aging, because of the success of antiretroviral therapy (ART) in extending life expectancy, and ongoing new diagnoses among older people [1–5]. ART has greatly reduced the occurrence of AIDS-defining illnesses. Most hospitalizations among PWH now result from non-AIDS-defining illnesses (NADIs), including liver, kidney and cardiovascular disease, and non-AIDS-defining cancers [6]. The clinical burden of NADIs among PWH is significant, and, because many NADIs are age-related, grows as the population ages [7]. Associated factors among PWH may increase rates of NADIs, including higher smoking rates than among the general population [8], comorbidities, and coinfection with other viruses [1]. HIV itself may also accelerate or accentuate some aspects of aging, leading to earlier occurrence of age-related diseases among PWH, although this remains controversial [5,9,10].

Whereas the CD4^+^ T-cell count has been used for decades to predict the risk of AIDS-defining illnesses [11], it is possible that the CD4^+^ : CD8^+^ T-cell ratio may be a useful measure to predict NADIs [12–15]. The ratio reflects both immune deficiency (decreased CD4^+^ T-cell count) and immune activation (elevated CD8^+^ T-cell count). A ratio greater than 1 is considered clinically normal [13]. A low CD4^+^ : CD8^+^ ratio is a marker of HIV infection. Despite substantial CD4^+^ T-cell recovery during the first years of ART [16], and gradual CD8^+^ count decline over time on ART, most PWH have a CD4^+^ : CD8^+^ ratio less than 1 even after many years of ART [14,15], reflecting incomplete immune recovery with ongoing immune activation and dysfunction [12,13]. Within the range of (generally low) CD4^+^ : CD8^+^ ratio values seen among PWH on ART, lower ratios have been associated with NADIs [14,15,17], pulmonary emphysema [18], frailty [19] and incident tuberculosis in Africa [20]. However non-AIDS mortality was not associated with CD4^+^ : CD8^ +^ ratio in the multicentre cohort study ART-CC (although AIDS-related mortality was) [21].

There is some evidence that the extent of CD4^+^ : CD8^+^ ratio recovery among PWH on ART reduces with age. A London study reported being over 50 at diagnosis was associated with reduced CD4^+^ : CD8^+^ recovery [22]. In the UK, people diagnosed with HIV after 50 are often diagnosed late, defined as having a CD4^+^ count below 350 cells/μl, because of delayed testing and more rapid CD4^+^ count decline after infection [3,23]. Late diagnosis is associated with reduced CD4^+^ : CD8^+^ recovery and other poorer outcomes [24,25]. One study found that over-50s had reduced CD4^+^ : CD8^+^ recovery even after adjusting for baseline CD4^+^[22]. In contrast, a Canadian study found no association between age and CD4^++^ : CD8^+^ ratio normalization (to >1) after adjusting for baseline CD4^+^ count [26].

The CD4^+^ : CD8^+^ ratio also declines with age in the general population, associated with a reduction in CD4^+^ T cells and an expansion of terminally differentiated CD8^+^ T cells, notably associated with cytomegalovirus (CMV) [27]. A Swedish general population study found that whereas 8% of individuals aged 20–59 had a CD4^+^ : CD8^+^ ratio less than 1, 16% of those aged 60–94 did [28]. A ratio less than 1 was associated with mortality among over-60s [28]. Two Swedish longitudinal studies of the very elderly (OCTO and NONA) reported that a CD4^+^ : CD8^+^ ratio less than 1 was part of an ‘immune risk profile’ associated with mortality [29,30]. However, this association was not found among elderly Belgian women in the BELFRAIL study, suggesting it varies across different contexts [27,31].

Here, a longitudinal dataset of people with HIV under care at the Royal Free London NHS Foundation Trust, London, UK, who initiated ART between 2001 and 2015 and achieved HIV-1 viral suppression, was used to investigate the association between age at ART initiation and long-term CD4^+^ : CD8^+^ recovery, adjusting for baseline CD4^+^ count, ethnicity, gender, and probable route of HIV transmission. The aim was to investigate the association of age at ART initiation with CD4^+^ : CD8^+^ ratio recovery among virally suppressed people on ART, and to characterize the potential for CD4^+^ : CD8^+^ recovery in this population by age.

## Methods

### Dataset and inclusion

Anonymized data were obtained from the Royal Free HIV Cohort Study (RFHCS), an observational database of people attending the HIV clinic at the Royal Free Hospital, London, UK. The sample included people meeting the following criteria: aged over 20 years at ART initiation; starting ART between 2001 and 2015; baseline CD4^+^ and CD8^+^ measurements taken within 6 months prior to starting ART; and viral suppression (viral load <1000 copies/ml) achieved within 6 months of starting ART. The database closed for analysis on 30 June 2019.

The period selected for initiating ART (2001 to 2015) allowed participants the potential for at least one annual measurement over 5 years (entrants from early 2015 had over 4 years follow-up). The viral load restriction enabled study of CD4^+^ and CD8^+^ trajectories under optimal treatment. Viral suppression is often defined as less than 50 copies/ml but it was decided that the WHO definition of less than 1000 copies/ml [32] was sufficient and more realistic over long-term treatment. If a viral load greater than 1000 copies/ml was recorded, follow-up was censored on that date. Individuals who left the study or died had their follow-up censored on that date.

The outcome was CD4^+^ : CD8^+^ ratio at 5 and 10 years after ART initiation. CD4^+^ : CD8^+^ ratio was calculated from CD4^+^ and CD8^+^ counts measured simultaneously during routine clinical practice. 92.6% of people achieved viral suppression (HIV-1 viral load <1000 copies/ml) within 6 months. Between 6 months and 5 years after starting ART, a median of 12 measurements per person was recorded (IQR 8–16, range 0–42), giving a median of 2.7 measurements per year. Measurements taken closest to the anniversary of the person's ART initiation were used. If no data was available within a +/−6-month window of this anniversary, a missing value was recorded. The resulting dataset included 1859 people at baseline.

### Demographic characteristics

Demographic variables included: age at initiating ART, gender, likely route of HIV transmission (MSM, heterosexual, and unknown or other transmission, including intravenous drug use), and ethnicity (white, black and other or unknown, see Table 3 footnote). As gender and route of HIV transmission are strongly correlated, these characteristics were captured using a single four-level composite variable (women, MSM, heterosexual men and men with other or unknown HIV transmission).

### Statistical analysis

All analyses were conducted using STATA MP version 17.0.

#### Descriptive statistics

Distributions of CD4^+^ counts, CD8^+^ counts and CD4^+^ : CD8^+^ ratio within the sample tended to be right skewed, so median values were used to summarize these variables across 10 years after ART initiation.

#### Multivariable linear regression

To capture the outcomes of long-term ART, 5 years was chosen a priori as the primary endpoint for regression analysis, with a secondary endpoint of 10 years for those with sufficient follow-up. Multivariable linear regression was used to investigate the association of age at baseline with CD4^+^ : CD8^+^ ratio after 5 and 10 years ART, while adjusting for baseline CD4^+^ count, calendar year at ART initiation, ethnicity, and gender/ route of HIV transmission, and censoring follow-up if viral load greater than 1000 copies/ml had occurred.

For the multivariable analysis, CD4^+^ : CD8^+^ ratios were logged (natural log) to normalize their distribution. Effect estimates obtained from the model were back-transformed to the original CD4^+^ : CD8^+^ ratio scale by taking the exponential, so that estimates could be interpreted as fold differences. For example, the coefficient value of 0.71 associated with baseline age 70–79 represents an adjusted CD4^+^ : CD8^+^ ratio 29% lower than the reference category of age 30–39.

As bivariate analysis suggested that age had a negative, nonlinear association with CD4^+^ : CD8^+^ ratio, age was considered in 10-year bands from baseline age 20–29 to 70–79 years. The 30–39 band was used as a reference group because they were a larger group than age 20–29, increasing precision of estimates.

Calendar year of ART initiation was included in the model because ART medication has improved over time [2]. Baseline CD4^+^ count (normalized by taking the square root) was included as a proxy for stage of disease at ART initiation, as this is known to be highly influential on long-term outcomes including CD4^+^ : CD8^+^ ratio recovery [2,15,25,33].

There was little evidence of multicollinearity or heteroscedasticity. There were more residual outliers than expected under normality. However, removing residual outliers (standardized residual deviance greater than 4, including four datapoints for year 5 and 1 datapoint for year 10) had little effect on independent variable coefficients, although it slightly increased the overall fit of the model. Thus, results presented below are for the full dataset.

## Results

### Demographic characteristics at start of antiretroviral therapy

The demographic composition of the sample reflects the two major HIV epidemics in London between 2001 and 2015. The largest number of HIV cases occurred among MSM. A second, heterosexual, epidemic was occurring mainly among men and women of Black African origin [4,34,35].

At baseline, there were 1392 men (75%) and 467 women. The percentage of men was higher with older age (Table 1, column 3).

**Table 1:** Sample sizes and censorship by age at baseline, year 5 and year 10 of antiretroviral therapy.

	Baseline		Year 5				Year 10^a^			
Age at ART initiation	Sample size	Percentage male	Sample size	Percentage censored within 5 years from all causes	Number (%) censored because of viral rebound	Number (%) who died within 5 years	Sample size	Percentage censored within 10 years from all causes	Number (%) censored because of viral rebound	Number (%) who died within 10 years
20–29	235	65.11	150	36.17	47 (20.00)	2 (0.85)	74	68.51	68 (28.94)	4 (1.70)
30–39	748	73.66	511	31.68	114 (15.24)	11 (1.47)	317	57.62	153 (20.45)	22 (2.94)
40–49	597	77.72	433	27.47	67 (15.47)	11 (1.84)	230	61.47	97 (16.25)	19 (3.18)
50–59	221	81.00	166	24.89	18 (8.14)	5 (2.26)	85	61.54	25 (11.31)	9 (4.07)
60–69	49	75.51	37	24.49	2 (4.08)	1 (2.04)	13	73.47	3 (6.12)	4 (8.16)
70–79	9	80.00	7	20.00	2 (22.2)	0	5	44.44	2 (22.2)	2 (22.22)
Total	1859	74.88	1304	29.85	250 (13.45)	30 (1.61)	724	61.05	396 (21.30)	60 (3.23)

Columns show, by age group (col 1): sample sizes at baseline (col 2), year 5 (col 4) and year 10 (col 8); percentage male at baseline (col 3); percentage censored because of all causes by year 5 (col 5) and year 10 (col 9); number (and percentage) censored because of viral load rebound (viral load >1000 HIV-1 copies/ml) by year 5 (col 6) and year 10 (col 10); number (and percentage) censored because of death by year 5 (col 7) and year 10 (col 11).

a Numbers and percentages for censorship by year 10 are cumulative, from baseline to year 10.

Among men, HIV transmission was via sex between men for 1018 (73%), heterosexually acquired for 314 (23%), and had other or unknown transmission routes including intravenous drug use for 4%. Among MSM, 827 (81%) were of white ethnicity. Among heterosexual men, 174 (55%) were of black ethnicity.

**Fig. 1:**
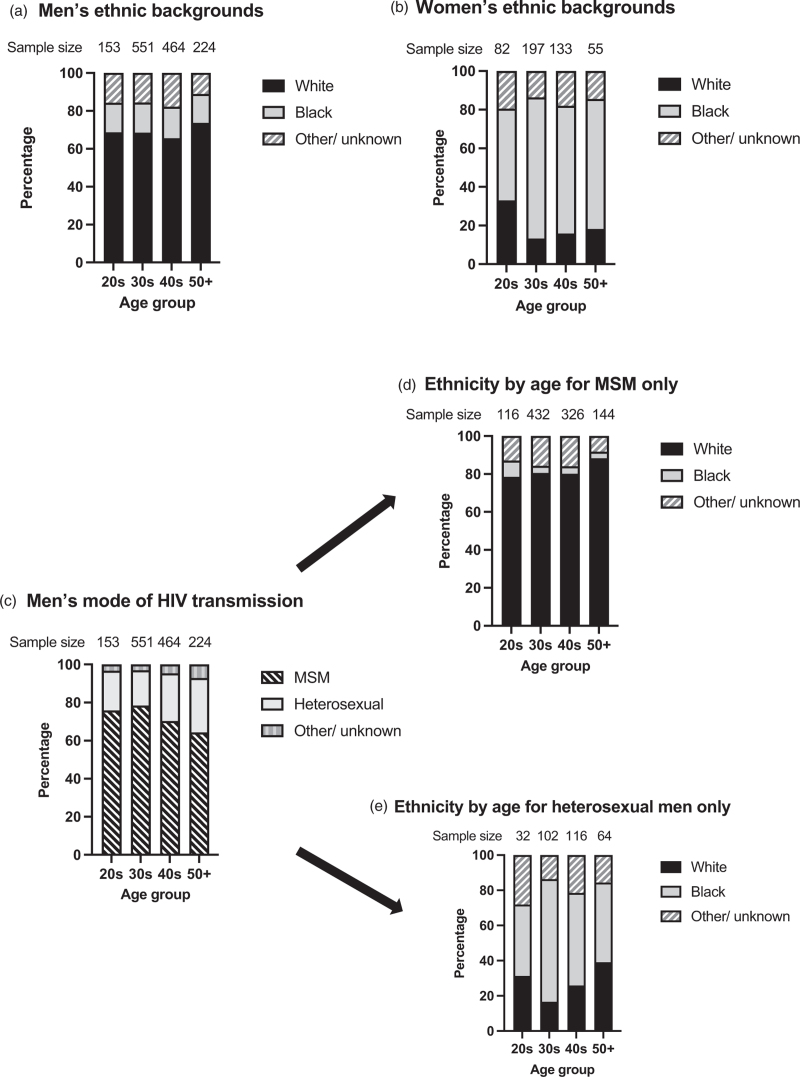
Demographic characteristics of the sample at baseline, by age.

HIV transmission was heterosexual for 97% of women. Among women, 308 (66%) were of black ethnicity (Fig. 1).

Columns show percentage of individuals within each category by age group. Sample sizes are shown above each column. (a) Men's ethnic backgrounds; (b) women's ethnic backgrounds. Black ethnicity includes Black African (*n* = 467) and Black Caribbean origin (*n* = 68). The ‘Other/unknown’ category includes Asian (*n* = 75), mixed race (*n* = 40), unspecified other ethnicity (*n* = 152) and unknown ethnicity (*n* = 26). (c) Men's route of HIV transmission. (d) Ethnic backgrounds of men with MSM HIV transmission only; (e) ethnic backgrounds of men with heterosexual HIV transmission only.

### Follow-up by age

Table 1 shows sample sizes by age at baseline, year 5 and year 10 after ART initiation. The percentage censored from all causes is shown, and the percentages censored specifically because of viral rebound (viral load >1000 HIV-1 copies/ml) and death.

The total proportion censored constituted 30% of the baseline sample by year 5 and 61% by year 10. The corresponding proportions censored because of viral rebound were 13 and 21%. There was a negative relationship between age at ART initiation and being censored from all causes within 5 years, partly because of a lower risk of viral rebound with older age. For example, the proportion of individuals with viral rebound by year 5 declined from 20% for age 20–29 to 4% for age 60–69, rising again after age 70 (2 of 9). There was a positive relationship between age at first ART and death during the next 10 years. Total deaths by year 10 of ART among under-60s were low, rising from 1.7% among age 20–29 to 8.2% among over-60s and 2 of 9 over-70s (Table 1).

### CD4^+^ T-cell counts, CD8^+^ T-cell counts, and CD4^+^ : CD8^+^ ratios over 10 years on antiretroviral therapy

**Fig. 2:**
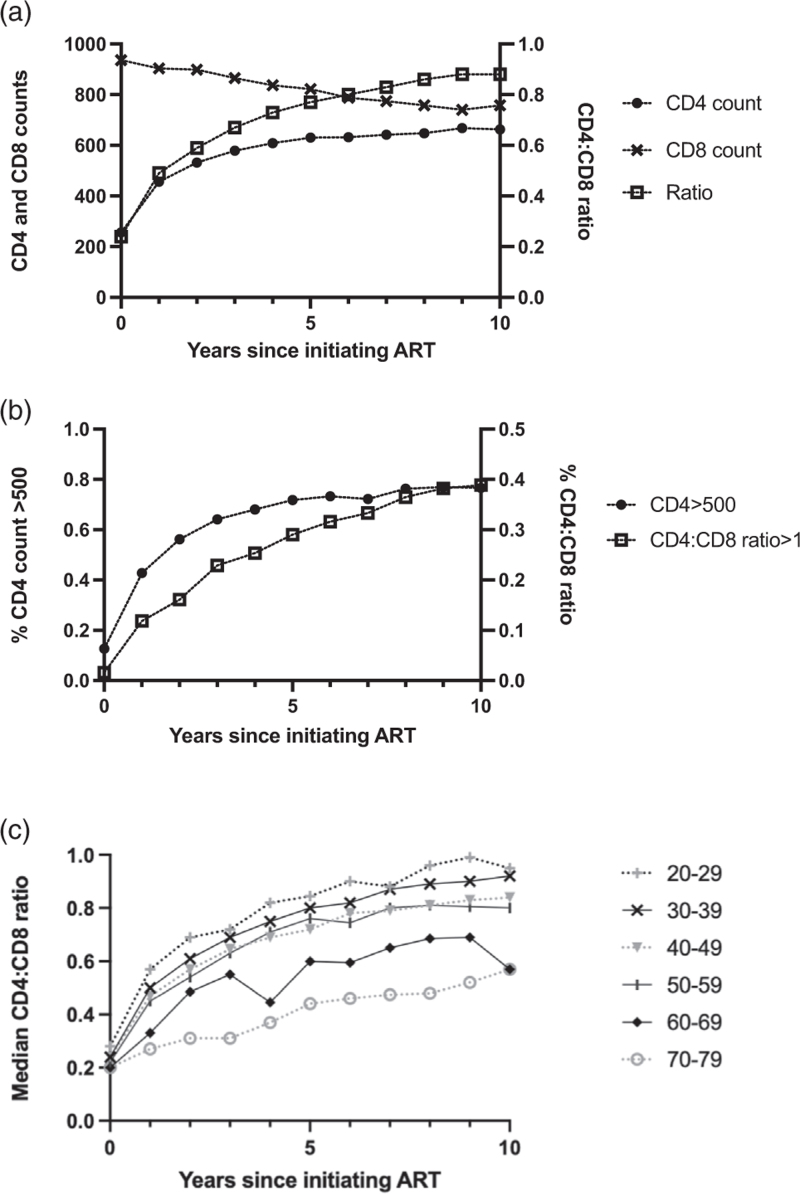
CD4^+^ T-cell count, CD8^+^ T-cell count and CD4^+^ : CD8^+^ ratio recovery across the whole sample over 10 years on antiretroviral therapy.

Table 2 and Fig. 2a show median CD4^+^ and CD8^+^ counts, and CD4^+^ : CD8^+^ ratio across 10 years after ART initiation in the sample overall and by baseline age. Overall, median CD4^+^ counts rose from 256 [interquartile range (IQR) 132–375] cells/μl at baseline to a maximum of 668 (IQR 511–837) in year 9. The largest increase occurred during the first 2 years. Median CD8^+^ count fell yearly from 936 (616–1365) at baseline to 741 (554–1032) in year 9, rising slightly in year 10. Median CD4^+^ : CD8^+^ ratio rose yearly from 0.24 (0.24, 0.38) at baseline to 0.88 in years 9 and 10 (at year 10 IQR = 0.64–1.17).

**Table 2:** Median CD4^+^ T-cell counts and CD4^+^ : CD8^+^ ratios, and percentage of people with CD4^+^ : CD8^+^ ratio greater than 1 by age at baseline, year 5 and year 10 of antiretroviral treatment.

Age at ART initiation	Baseline	Year 5	Year 10
	*Median CD4* ^*+*^ * T-cell counts (IQR)*		
20–29	271 (160–400)	653 (500–808)	636 (494–812)
30–39	255 (134–368)	642 (491–824)	681 (523–864)
40–49	255 (134–368)	623 (458–803)	679 (489–848)
50–59	261 (126–375)	634 (452–782)	623 (457–829)
60–69	268 (57–389)	556 (443–674)	544 (436–667)
70–79	134 (43–199)	549 (415–608)	621 (548–681)
Total	256 (132–375)	630 (479–810)	663 (507–846)
	*Median CD4* ^*+*^ * : CD8* ^*+*^ * ratios (IQR)*		
20–29	0.28 (0.18–0.42)	0.85 (0.63–1.08)	0.95 (0.75–1.29)
30–39	0.24 (0.14–0.36)	0.8 (0.59–1.06)	0.92 (0.71–1.2)
40–49	0.24 (0.14–0.36)	0.72 (0.55–0.99)	0.84 (0.61–1.09)
50–59	0.22 (0.12–0.39)	0.76 (0.5–1.11)	0.80 (0.56–1.24)
60–69	0.2 (0.11–0.40)	0.6 (0.41–1.04)	0.57 (0.46–0.86)
70–79	0.2 (0.14–0.27)	0.44 (0.38–0.54)	0.57 (0.52–0.60)
Total	0.24 (0.14–0.38)	0.77 (0.56–1.05)	0.88 (0.64–1.17)
	*% CD4* ^*+*^ * : CD8* ^*+*^ * ratio >1 (95% CI)*		
20–29	0.9 (0.1–3.0)	37 (29–45)	43 (32–55)
30–39	1.5 (0.7–2.6)	29 (25–33)	43 (37–49)
40–49	2.4 (1.3–3.9)	25 (21–29)	33 (27–39)
50–59	1.4 (0.28–3.9)	33 (26–41)	41 (31–52)
60–69	0 (0–7.4)^a^	27 (14–44)	23 (5–54)
70–79	0 (0–34)^a^	14 (0.36–58)	0 (0–52)^a^
Total	1.6 (1.1–2.3)	29 (26–31)	39 (35–42)

CI, confidence intervals; IQR, interquartile range.

a One-sided 97.5% CI.

Sample sizes at baseline, year 5 and year 10 were shown in Table 1. (a) Median values for CD4^+^ T-cell count, CD8^+^ T-cell count and CD4^+^ : CD8^+^ ratio. (b) Percentages of people with clinically normal CD4^+^ T-cell count (>500) and CD4^+^ : CD8^+^ ratio (>1.0). (c) Median values for CD4^+^ : CD8^+^ ratio over 10 years on ART, stratified by age at baseline.

Most people recovered a clinically normal CD4^+^ count within 2 years of starting ART but did not achieve a clinically normal CD4^+^ : CD8^+^ ratio over 10 years of treatment. The percentage of people with clinically normal CD4^+^ counts (>500 cells/μl) rose from 13% at baseline to 56% in year 2, 72% at year 5 and 77% at year 10. In contrast, the percentage of people with clinically normal CD4^+^ : CD8^+^ ratios (>1) rose yearly from 1.6% at baseline to just 38.8% in year 10 (Table 2 and Fig. 2b).

The percentage of people with normal CD4^+^ : CD8^+^ ratios (>1.0) at baseline, year 5 and year 10 was lower with older age, as were median CD4^+^ : CD8^+^ ratios. Baseline CD4^+^ counts were similar across ages 20–69 years (median values ranged from 255 to 271) but were lower among ages 70–79 (median 134). Among baseline ages 20–39 years, 43% achieved a ratio greater than 1.0 after 10 years ART, compared with 23% aged 60–69 and none aged 70–79. Most people in all age groups, including over-70s, achieved a clinically normal CD4^+^ count (>500 cell/μl) by year 5 of virally suppressive ART. However, in no age group did most people achieve a clinically normal CD4^+^ : CD8^+^ ratio (>1) over 10 years with viral suppression (Table 2).

Fig. 2c shows CD4^+^ : CD8^+^ ratio recovery over 10 years on ART stratified by age. Median CD4^+^ : CD8^+^ ratio increased in every age group, although none achieved a median ratio greater than 1 within 10 years. There was an association of older age with a lower CD4^+^ : CD8^+^ ratio from year 1 onward. Those in their 70s at baseline had substantially lower CD4^+^ : CD8^+^ ratios than those in their 60s. Therefore, these age groups were analysed separately despite the small sample size of the oldest age group.

### Multivariable regression results

Table 3 shows results of a multivariable linear regression model with the CD4^+^ : CD8^+^ ratio at years 5 and 10 of ART as the dependent variable, and age, gender/transmission, ethnicity, baseline CD4^+^ and year as the independent variables. Results for CD4^+^ : CD8^+^ ratio at years 5 and 10 of ART are shown. Results show fold changes in CD4^+^ : CD8^+^ ratio associated with each age category, relative to people starting ART aged 30–39 (the reference category). Fold changes below 1 indicate a reduced CD4^+^ : CD8^+^ ratio relative to those in their 30s.

**Table 3:** Association of age at baseline (first antiretroviral treatment) and CD4^+^ : CD8^+^ ratio at years 5 and 10 of antiretroviral therapy, adjusted for gender, probable route of HIV transmission, ethnicity, baseline CD4^+^ T-cell count and calendar year: results of a linear regression model.

	Year of ART					
	Year 5			Year 10		
	Fold difference	95% CI	*P*	Fold difference	95% CI	*P*
Baseline age						
20–29	0.98	0.90–1.06	0.579	0.98	0.87–1.10	0.70
30–39 (reference)	1.00			1.00		
40–49	0.94	0.87–0.98	0.014	0.95	0.87–1.02	0.140
50–59	0.91	0.84–0.99	0.026	0.95	0.85–1.06	0.37
60–69	0.83	0.71–0.97	0.017	0.82	0.66–1.07	0.15
70–79	0.71	0.50–1.00	0.052	0.62	0.42–0.94	0.024
Gender and route of HIV transmission						
Women (reference)	1.00			1.00		
Men, MSM	0.81	0.75–0.87	<0.001	0.72	0.65–0.80	<0.001
Men–heterosexual	0.85	0.78–0.92	<0.001	0.82	0.73–0.91	<0.001
Men, other or unknown transmission	0.95	0.80–1.13	0.561	0.87	0.67–1.13	0.287
Ethnicity						
White (reference)	1.00			1.00		
Black^a^	0.94	0.87–1.02	0.150	0.91	0.82–1.01	0.080
Other or unknown^b^	0.93	0.86–1.00	0.058	0.90	0.81–0.99	0.030
Baseline CD4^+^ T-cell count	1.04	1.03–1.04	<0.001	1.03	1.02–1.04	<0.001
Calendar year	1.02	0.99–1.06	0.161	0.99	0.93–1.05	0.657
Overall R^2^		0.23			0.06	
Sample size		1304			724	

Column 1 shows the predictor variables, including: age at first ART grouped into 10-year bands, with people who started ART in their 30s as the reference group; a four-level variable combining gender plus route of HIV transmission, with women as the reference group; ethnicity, with white as the reference group; CD4^+^ cell count at baseline; and baseline calendar year. The fold difference (or exponentiated coefficient) for each predictor variable is shown, with 95% confidence intervals and associated probability. The two bottom rows show sample sizes and the overall *R*^2^ for year 5 and year 10 of ART. Dependent variable is CD4^+^ : CD8^+^ ratio.

a Includes Black African (467 people) and Black Caribbean (68 people).

b Includes mixed ethnicity (40 people), Indian, Pakistani or Bangladeshi (24 people) other Asian (51 people), other ethnicity with no further information (152 people) and unknown ethnicity (26).

Five years after ART initiation, all age groups over 40 had lower CD4^+^ : CD8^+^ ratios than age group 30–39. At year 5 of ART, coefficient values indicate a graded relationship of older age with lower CD4^+^ : CD8^+^ ratio. At year 10 of ART, a similar pattern of association of older age with lower ratio was seen among coefficient values but was only statistically significant among over-70s.

Compared with women, both MSM and heterosexual men had a reduced CD4^+^ : CD8^+^ ratio at 5 and 10 years after ART initiation. Compared with the reference white ethnic group, people of Black and ‘other or unknown’ ethnicity showed some evidence of lower CD4^+^ : CD8^+^ ratio at 5 and 10 years.

Baseline CD4^+^ count had a highly significant positive association with CD4^+^ : CD8^+^ ratio at years 5 and 10. This is consistent with previous reports that CD4^+^ nadir is strongly associated with recovery, but also expected because CD4^+^ count is a component of CD4^+^ : CD8^+^ ratio. When baseline CD4^+^ count was included in the model, there was no evidence that CD4^+^ : CD8^+^ ratio at years 5 and 10 was related to calendar year of starting ART. If baseline CD4^+^ count was excluded from the model, calendar year was significant, but the association between CD4^+^ : CD8^+^ ratio and other predictor variables remained broadly similar (results not shown).

## Discussion

In a large sample of PWH, attending a London clinic, who maintained viral suppression on long-term ART, older age was associated with progressively reduced CD4^+^ : CD8^+^ recovery compared with people starting ART in their 30s. Older age was associated with lower CD4^+^ : CD8^+^ levels after initiating ART, and a lower percentage achieved a CD4^+^ : CD8^+^ ratio >1 after 5 and 10 years on ART.

After 5 years on ART, among people aged 40–59 at baseline, there was a moderately lower CD4^+^ : CD8^+^ ratio compared with those in their 30s at baseline. The CD4^+^ : CD8^+^ ratio was substantially lower among over-60s compared with people in their 30s at baseline. This difference was even larger in over-70s, albeit among a small sample.

After 10 years on ART, there remained a significantly lower CD4^+^ : CD8^+^ ratio among the 70–79-year-old group compared with the 30–39-year-old group. A graded reduction in ratio recovery with age was also seen among other age groups, but the differences were not statistically significant. This could be because of lack of power, as sample size declined over the 10-year study period (Table 1). However, an alternative explanation suggested by our results is that whilst CD4^+^ : CD8^+^ recovery after ART initiation is slower at older ages, after 10 years ART, the CD4^+^ : CD8^+^ ratio of people aged 40–59 years at baseline approaches that of people aged 30–39 years at baseline (Table 3).

It should be emphasized that we focussed on outcomes under near-optimal treatment conditions. The sample only included people on ART who achieved viral suppression within 6 months and maintained it. Estimates presented here are not intended to represent the whole HIV-positive population.

The onset of substantial age-related reduction in CD4^+^ : CD8^+^ recovery following ART initiation occurred after age 60 years, older than in a previous London study [22] which reported that being diagnosed with HIV aged more than 50 years was associated with reduced recovery. In contrast, we report that virally suppressed people aged 50–59 years had very similar CD4^+^ : CD8^+^ ratios to people aged 40–49. These differences may arise from the longer treatment period in this study (5 or 10 years) compared with a median treatment period of 38 months in the previous study [22]. Evidence presented in Table 3 suggests that over very long-term treatment, people aged from 40 to 59 years at baseline almost caught up with younger age groups in terms of CD4^+^ : CD8^+^ ratio recovery. Another possible reason for our different results is that we divided over-50 s by 10-year age bands, enabling us to elucidate differences in CD4^+^ : CD8^+^ ratios between people in their 50s, 60s and 70s at ART initiation.

Our results also differ from a Canadian study [26], which reported that once baseline CD4^+^ count was included in a multivariable model, there was no association of age and CD4^+^ : CD8^+^ ratio normalization to 1 or above; this study also grouped over-50s in a single age group.

The strong association we found between baseline CD4^+^ count and CD4^+^ : CD8^+^ ratio after 5 and 10 years on ART (Table 3) is consistent with previous reports that CD4^+^ nadir is highly influential on outcomes even after many years on ART [1,2,25,26,33,36]. However, as CD4^+^ count is a component of the CD4^+^ : CD8^+^ ratio, these two variables were expected to be mathematically correlated, and we should not over-interpret these coefficients.

Baseline CD4^+^ count is a proxy for stage of HIV disease progression at the start of treatment, so associations with age in multivariable analysis shown in Table 3 somewhat account for later diagnoses among older people. Ethnic differences in baseline CD4^+^ count should also be accounted for in the multivariable model but it is possible that some residual confounding remains.

The absence of association between calendar year at starting ART and CD4^+^ : CD8^+^ ratio recovery, when baseline CD4^+^ was adjusted for, was unexpected, given improvements in ART medications from 2001 to 2019, over and above earlier initiation of ART. Possibly such effects might have been attenuated in a virally suppressed population whose treatment was nearly optimal.

Lower CD4^+^ : CD8^+^ ratio associated with being male is consistent with previous findings [13,14,28]. The negative effects on CD4^+^ : CD8^+^ ratio of being male (particularly MSM) relative to women, and of nonwhite ethnicity relative to white ethnicity, tended to increase between years 5 and 10 on ART.

Across age groups, most people did not achieve a CD4^+^ : CD8^+^ ratio greater than 1 during long-term ART, although most people in all age groups achieved a CD4^+^ count greater than 500 cells/μl within 5 years of starting ART (Fig. 2). This is consistent with previous reports that CD4^+^ recovery is generally not followed by CD4^+^ : CD8^+^ ratio recovery to clinically normal levels [12–15].

The study had limitations. Sample sizes for all ages were much reduced after 10 years of ART. In particular, numbers of people aged more than 60 years in the sample are small, so results should be interpreted with caution. However, older PWH are an under-studied but growing population, with a high health burden from NADIs, so these data have value for characterizing older people's potential for CD4 : CD8^+^ recovery despite remaining uncertainties. As more data from HIV-positive over-60s become available, it should become possible to further characterize the age-associated reduction in CD4 : CD8^+^ recovery, including different rates of response to treatment: for example, to investigate whether younger (baseline ages 20–39) and middle-aged (ages 40–59) people eventually converge at a similar CD4 : CD8^+^ ratio over very long-term ART.

Age-related mortality (Table 1) raises the possibility of survivor bias, with survivors healthier than the baseline population. As deaths among under-60s were low, this would tend to affect older people. It would probably attenuate the negative relationship between age and CD4^+^ : CD8^+^ recovery so, if anything, the age effect may be underestimated. A positive relationship between older age and maintenance of viral suppression, resulting from ART adherence, is consistent with previous findings [37].

We lacked data on CMV coinfection, so could not examine its effects on CD4^+^ : CD8^+^ ratio recovery, a topic for future research. We note that Mussini *et al.* in 2015 had CMV data for a subset of their sample, enabling them to compare the CD4^+^ : CD8^+^ ratio and clinical endpoints among CMV+ and CMV− PWH: they reported no difference between these groups.

To conclude, we report that, among a virally suppressed HIV positive London population, CD4^+^ : CD8^+^ ratio recovery over long-term ART lessened with older age at ART initiation. A substantial decline in ratio recovery occurred after age 60 years. People aged 40–49 and 50–59 at baseline had similar trajectories of CD4^+^ : CD8^+^ ratio recovery.

## Acknowledgements

Concept and design of this study, statistical analysis and writing were undertaken by C.J.S., C.J.H., and F.L. M.J.. F.M.B. and S.K.-d.L. are part of the clinical research team and contributed to the clinical interpretation of the results and commented on early drafts of the manuscript. Data management was by C.C.

Ethical approval for the Royal Free HIV Cohort Study was obtained from the London South-East Research Ethics Committee (REC reference 19/LO/0091).

### Conflicts of interest

There are no conflicts of interest.
